# Supplementary material for: Real-World Outcomes in Patients with Metastatic Colorectal Cancer in Spain: The RWD-ACROSS Study
Source: Cancers (Basel). 2023 Sep 17;15(18):4603. doi: 10.3390/cancers15184603 (PMC10526223; doi:10.3390/cancers15184603)
Supplement: Supplementary file 1 [file cancers-15-04603-s001.zip › cancers-2492494-supplementary.pdf]

Real-world outcomes in patients with metastatic colorectal cancer in Spain: the RWD-ACROSS study

Carles Pericay [1]*, Ana Fernández Montes [2], Vicente Alonso Orduña [3], Ismael Macias Declara [4], Elena Asensio Martínez [5], Nuria Rodríguez Salas [6], Esperanza Torres [7], Diego Cacho Lavín [8], Rosa María Rodríguez Alonso [9], Esther Falcó [10], Joan Carles Oliva [4] and Lluis Cirera [1]

*Correspondence: cpericay@gmail.com; Tel.: +34 648874982; ORCiD: 0000-0002-4975-7851

Supplementary materials

Table S1. Specific primary tumour locations.

Location	Number (%)
**Right-sided**	**534 (26.7)**
Caecum	200 (10.0)
Ascending colon	171 (8.5)
Hepatic flexure	70 (3.5)
Transverse colon	93 (4.6)
**Left-sided**	**1444 (72.1)**
Splenic flexure	73 (3.6)
Descending colon	100 (5.0)
Sigmoid colon	586 (29.3)
Rectus sigma	197 (9.8)
Rectum	488 (24.4)
**Both sides**	**24 (1.2)**
**Total**	**2002 (100.0)**

Table S2. Overall survival according to the sequence of first- and second-line therapies in 1428 patients who received second-line therapy. 'Biological therapy' means treatment with either an anti-VEGF or an anti-EGFR agent in addition to chemotherapy.

First-line therapy	Second-line therapy	Median OS (months)	Median OS (months) by tumour location and RAS mutation status			
			Left-sided		Right-sided	
			Wild-type *RAS*[1]	Mutant *RAS*[2]	Wild-type *RAS*[3]	Mutant *RAS*[4]
Any treatment	Any treatment	(n=1428) 29.21 (27.89–30.52)				
Chemotherapy only	Chemotherapy only	(n=369) 25.54 (22.85–28.22)	(n=63) 34.36 (23.62–45.09)	(n=141) 27.93 (22.73–33.13)	(n=23) 24.78 (15.85–33.71)	(n=49) 19.96 (12.72–27.20)
Anti-VEGF	Anti-VEGF	(n=286) 30.98 (28.18-33.78)	(n=44) 36.06 (28.02–44.10)	(n=150) 30.98 (27.62–34.34)	(n=9) 22.42 (10.83–34.01)	(n=51) 28.09 (21.40–34.79)
Anti-VEGF	Anti-EGFR	(n=72) 25.41 (22.87–27.94)	(n=47) 26.52 (23.84–29.20)	–	(n=15) 18.62 (13.44–23.79)	–
Anti-EGFR	Anti-VEGF	(n=130) 31.93 (28.11–35.75)	(n=80) 33.93 (26.64–41.22)	–	(n=24) 21.63 (12.30–30.97)	–

Values are presented as median (95% confidence interval) months unless stated otherwise.
[1] *P*=0.616.
[2] *P*=0.808.
[3] *P*=0.039.
[4] *P*=0.241.
EGFR, endothelial growth factor receptor; OS, overall survival; VEGF, vascular endothelial growth factor.

Figure S1. CONSORT flow chart describing mCRC patients screened in the RWD-ACROSS study by line of therapy and mutation status.

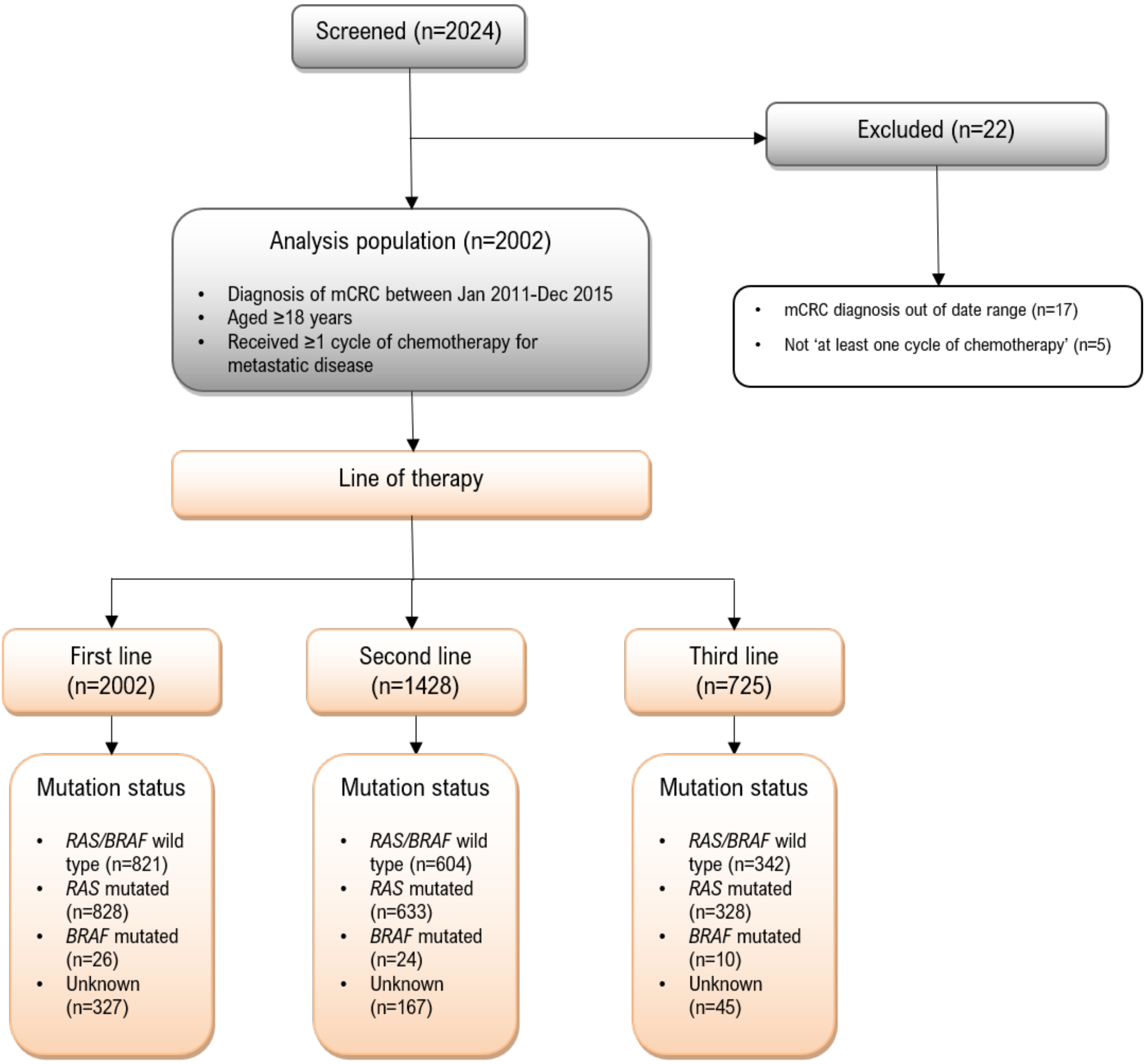

Figure S2. Kaplan-Meier estimate of overall survival probability following (**a**) first-line, (**b**) second-line, and (**c**) third-line systemic treatment.

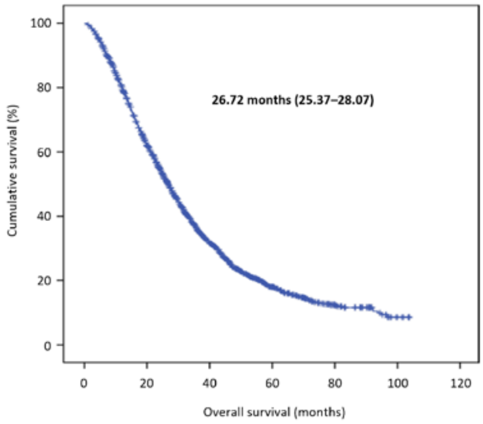

(**a**)

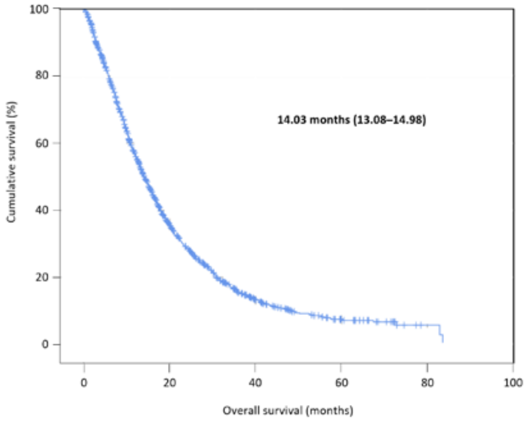

(**b**)

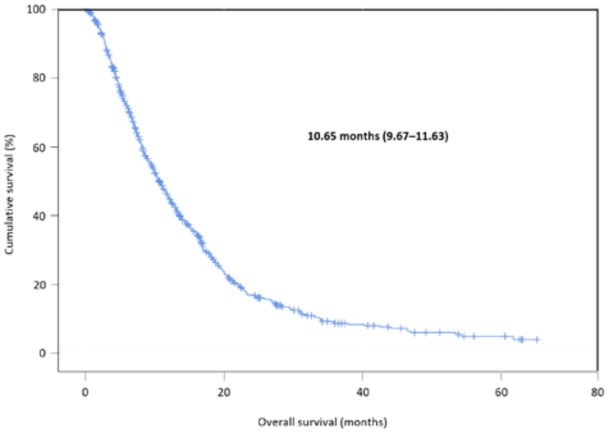

(**c**)

Figure S3. Kaplan-Meier estimate of progression-free survival probability following (**a**) first-line, (**b**) second-line, and (**c**) third-line systemic treatment.

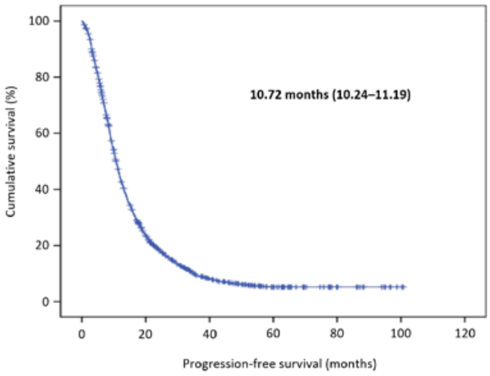

(**a**)

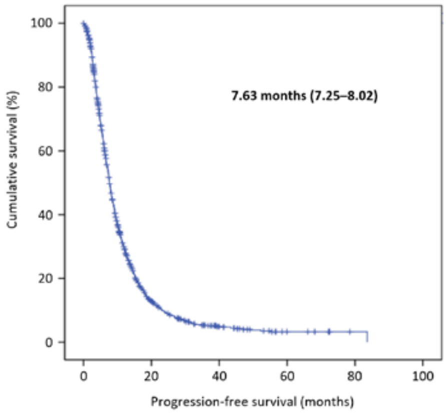

(**b**)

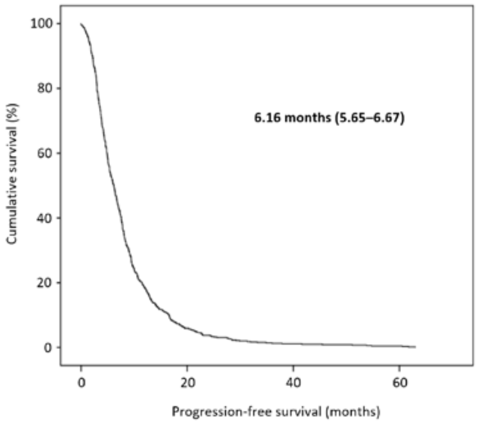

(**c**)
